# Molecular prevalence and risk factors of ovine herpesvirus-2–associated malignant catarrhal fever in cattle from Riau Province, Indonesia: A cross-sectional nested polymerase chain reaction study

**DOI:** 10.14202/vetworld.2026.554-568

**Published:** 2026-02-17

**Authors:** Annisa Yohanes, Dyah Ayu Widiasih, Agus Wiyono, Rini Damayanti, Faralinda Sari, Dewi Anggreini

**Affiliations:** 1 Postgraduate Student, Postgraduate Program, Faculty of Veterinary Medicine, Universitas Gadjah Mada, Yogyakarta, Indonesia; 2 Department of Veterinary Public Health, Faculty of Veterinary Medicine, Universitas Gadjah Mada, Yogyakarta, Indonesia; 3 Research Center for Veterinary Science, National Research and Innovation Agency (BRIN), Bogor, Indonesia; 4 Animal Husbandry and Animal Health Services of Riau Province, Riau, Indonesia

**Keywords:** cattle, cross-sectional study, malignant catarrhal fever, nested PCR, ovine herpesvirus 2, prevalence, risk factors, sheep

## Abstract

**Background and Aim:**

Malignant catarrhal fever (MCF) is a highly fatal disease caused by *ovine herpesvirus-2* (OvHV-2), with sheep acting as asymptomatic reservoir hosts. In Indonesia, MCF remains underdiagnosed due to its predominantly subclinical presentation, particularly in regions where mixed ruminant farming is practiced. Riau Province has a large population of Bali cattle, and sheep production is increasing, raising concerns regarding OvHV-2 spillover. This study aimed to determine the molecular prevalence of OvHV-2 infection in cattle and identify individual- and herd-level risk factors associated with the occurrence of MCF in Riau Province, Indonesia.

**Materials and Method:**

A cross-sectional study was conducted between August and December 2024 involving 274 beef cattle from 76 farms across five districts of Riau Province. Buffy coat DNA extracted from ethylenediaminetetraacetic acid-anticoagulated blood samples was examined for OvHV-2 using nested polymerase chain reaction (PCR). Epidemiological data were collected through structured questionnaires administered to farmers. The associations between potential risk factors and OvHV-2 positivity were evaluated using chi-square tests and multivariate logistic regression analysis.

**Result:**

OvHV-2 DNA was detected in 33 of 274 cattle, yielding an individual-level prevalence of 12.0% (95%CI: 8.0–16.0%). At the farm-level, 21 of 76 farms (27.6%; 95% confidence interval [CI]: 17.0%–38.0%) had at least one PCR-positive animal. Most infected cattle (n = 32/33) were clinically asymptomatic, emphasizing the silent nature of infection. The multivariable analysis identified cattle aged 2–5 years as having a significantly lower risk of infection (adjusted odds ratio [aOR] = 0.239; p = 0.004). In contrast, farms that practiced mixed rearing of cattle and sheep showed a markedly increased risk of MCF (aOR = 8.218; p = 0.015), as did farms located within 100 m of sheep holdings (aOR = 4.083; p = 0.027).

**Conclusio:**

OvHV-2 infection is endemic but largely subclinical in cattle in Riau Province. Close proximity to sheep and co-rearing practices are the primary drivers of MCF risk. These findings underscore the importance of molecular surveillance and the implementation of biosecurity measures, particularly spatial separation between cattle and sheep, to prevent OvHV-2 transmission and reduce economic losses in susceptible cattle populations.

## INTRODUCTION

Riau Province is located on the island of Sumatra, Indonesia. According to Government Regulation Number 72 of 2019, which came into effect on October 8, 2019, Riau covers an area of 87,023.66 km², extending from the slopes of the Bukit Barisan mountain range to the Malacca Strait, within coordinates 1°05’00”S–2°25’00”N latitude and 100°00’00”E–105°05’00”E longitude. The principal large-ruminant livestock commodity in the province is *Bos javanicus* (Bali cattle).

In addition to Bali cattle, small ruminants are commonly reared in Riau Province. The increasing population of sheep and goats, together with predominantly free-range management systems, heightens the risk of malignant catarrhal fever (MCF) outbreaks. In recent years, numerous clinical cases resembling Jembrana disease have been reported in Bali cattle. However, polymerase chain reaction (PCR) testing conducted at the UPT LVKH between 2021 and 2023 largely yielded negative results for Jembrana disease virus. Given the clinical similarities between the two conditions, it is suspected that many of these Jembrana-negative cases may in fact represent MCF infections.

MCF is a frequently fatal disease affecting large ruminants, including water buffalo, cattle, deer, and gaur, and is caused by viruses belonging to the subfamily Gammaherpesvirinae [1, 2]. Transmission of *ovine herpesvirus-2* (OvHV-2) from sheep to cattle occurs primarily via aerosol inhalation of virus-containing nasal secretions from infected carrier sheep [3]. In susceptible large ruminants such as cattle, deer, bison, water buffalo, and pigs, OvHV-2 causes clinically apparent sheep-associated MCF (SA-MCF), whereas domestic sheep remain asymptomatic carriers [4]. The disease may present in a peracute form without obvious clinical signs, resulting in sudden death, or as a more prolonged illness lasting one week or longer, in which survival may occur. Peracute disease is most commonly observed in highly susceptible hosts, particularly deer species, with death occurring within 12–24 h following depression, weakness, diarrhea, or dysentery [5]. The high fatality rate associated with MCF leads to substantial economic losses. In Riau Province, the introduction and expansion of sheep populations, which serve as reservoirs of OvHV-2, pose a considerable risk for disease spread and associated economic impacts due to cattle mortality and control measures. Similar sporadic outbreaks have been reported in Bali and East Nusa Tenggara, often resulting in severe losses among Bali cattle, which are of major economic importance to smallholder farmers. Such outbreaks disrupt cattle production systems and impose significant financial burdens, thereby threatening household income and food security [6].

Recent evidence from Southeast Asia indicates that OvHV-2-associated MCF remains endemic in several livestock-producing regions, although published prevalence data from 2022 to 2024 are limited. In Malaysia, confirmed outbreaks in Bali cattle have been documented on mixed farms where cattle and sheep are reared in close proximity, with affected animals showing classical clinical signs such as fever, ocular discharge, and corneal opacity. In Indonesia, sporadic cases continue to be reported from Bali, Java, Nusa Tenggara Timur (including Timor), and Sumatra, suggesting sustained circulation of OvHV-2 in regions characterized by mixed-ruminant production systems. Although recent empirical prevalence data from Thailand and Timor are scarce, similar ecological conditions and husbandry practices in these areas suggest that MCF is likely to occur wherever sheep or goats co-graze with susceptible cattle. Collectively, these observations emphasize that MCF remains an underdiagnosed yet persistent threat in Southeast Asia, particularly in settings involving mixed small- and large-ruminant farming [7, 8].

From an epidemiological perspective, two main forms of MCF are recognized: wildebeest-associated MCF (WA-MCF), caused by alcelaphine herpesvirus-1 (AHV-1), and SA-MCF, caused by OvHV-2. These two forms are clinically and pathologically indistinguishable [9]. Cattle adapted to tropical and subtropical environments are considered more susceptible to MCF than buffalo (*Bubalus bubalis*). The reported susceptibility hierarchy is Bali cattle, crossbred Bali cattle, water buffalo, *Bos indicus*, and *Bos taurus* [10]. MCF remains a significant problem in Indonesia because of its sporadic yet often lethal nature and its tendency to cause outbreaks, particularly in areas where Bali cattle are grazed in proximity to sheep. Accordingly, we hypothesized that cattle raised near sheep flocks have a higher risk of OvHV-2 infection due to environmental exposure.

Despite the well-documented lethality and economic significance of MCF in susceptible ruminants, critical gaps remain in understanding its epidemiology in Indonesia, particularly at the provincial and production system levels. Existing studies are largely limited to outbreak reports or isolated case investigations, with minimal emphasis on systematic molecular surveillance of apparently healthy cattle. In Riau Province, where Bali cattle dominate livestock production and small ruminant populations are increasing, robust data on the molecular prevalence of OvHV-2 in cattle are scarce. Furthermore, the influence of local husbandry practices, including free-range management, mixed ruminant rearing, and spatial proximity between cattle and sheep, has not been adequately quantified. The frequent occurrence of Jembrana disease–like clinical cases that test negative by PCR further suggests potential misclassification and underrecognition of MCF. The absence of region-specific, risk factor–oriented epidemiological evidence limits accurate risk assessment and constrains the development of targeted biosecurity and disease control strategies. Addressing these gaps requires integrated molecular and epidemiological investigations tailored to local farming systems.

The aim of this study was to determine the molecular prevalence of OvHV-2 infection in cattle across multiple districts of Riau Province, Indonesia, using nested PCR. In addition, this study aimed to identify and evaluate individual-level and herd-level risk factors associated with OvHV-2 infection, with particular emphasis on cattle age, management practices, and cattle–sheep proximity. By generating province-specific baseline epidemiological data, this study seeks to improve understanding of MCF transmission dynamics and to support evidence-based surveillance, prevention, and control strategies in mixed-ruminant production systems.

## MATERIALS AND METHODS

### Ethical approval

All study procedures were approved by the Ethical Committee of the National Research and Innovation Agency. Ethical clearance was granted on March 5, 2024, under approval number No. 048/KE.02/SK/03/2024. The study involved cattle owned by farmers from five regions of Riau Province, Indonesia. A collection of biological samples was carried out with informed consent obtained from all participating farmers.

### Study period and location

**Figure 1:**
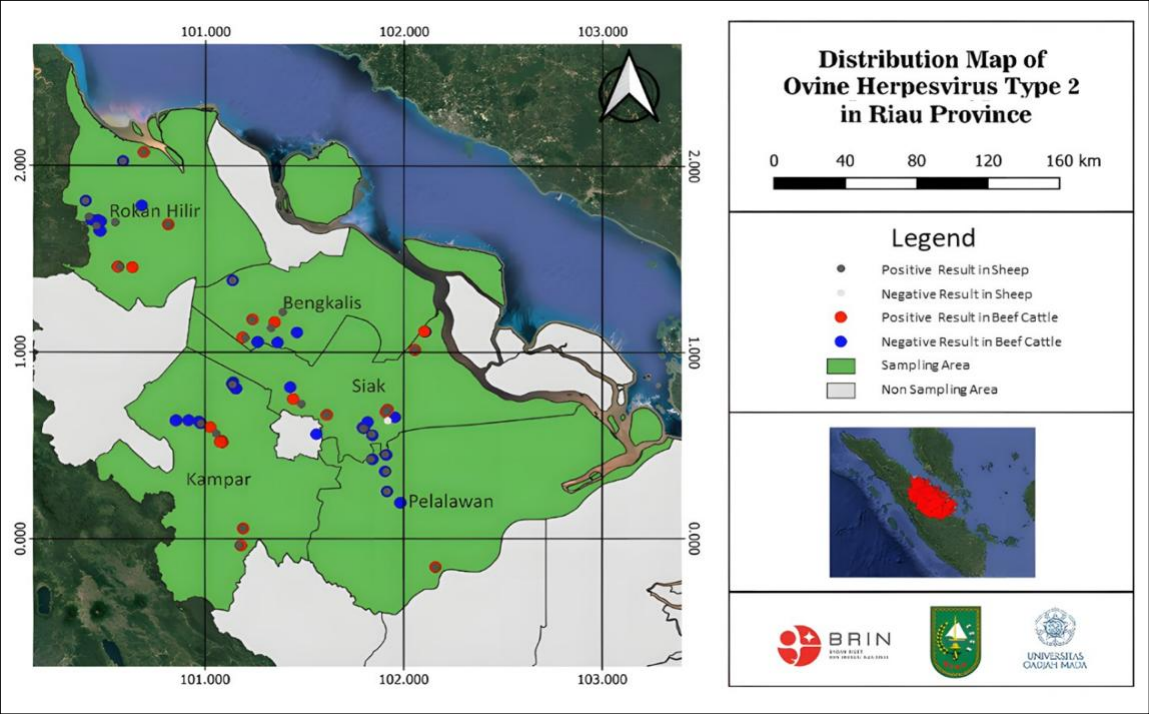
Geographic location of Riau Province, Indonesia, showing the distribution of sampled districts included in the malignant catarrhal fever surveillance study.

A cross-sectional study was conducted between August and December 2024 across five regencies in Riau Province: Pelalawan, Siak, Kampar, Rokan Hilir, and Bengkalis. Figure 1 shows the distribution of sampled districts included in the malignant catarrhal fever monitoring study in Riau Province.

### Study design

This study investigated the presence of MCF based on clinical examinations of Bali cattle, followed by molecular identification of the infectious agent using PCR. Blood samples were collected in anticoagulant tubes, and buffy coat fractions were subsequently isolated for laboratory analysis.

The primary objective was to estimate the prevalence of MCF in cattle. A total of 274 cattle were sampled from 76 breeding farms. Sample size determination was based on the epidemiological formula, n = 4PQ/L² [46], using an assumed prevalence of 54% derived from a preliminary study conducted in 2021. That earlier investigation, carried out collaboratively by the Riau Province Livestock and Animal Health Service, the Veterinary Research Center, the Health Research Organization, and the National Research and Innovation Agency, reported that 54% of 50 sheep blood samples collected from five areas were positive for MCF. With a margin of error of 5%, the calculated sample size was 398.

Based on this calculation, 265 samples (two-thirds of 398) were allocated for whole bovine blood samples, while 133 samples (one-third of 398) were allocated for sheep mucosal swab samples. To compensate for potential sample loss and enhance representation across regencies, the final number of collected samples was increased to 274 cattle and 159 sheep mucosal swabs. Whole blood samples anticoagulated with ethylene-diaminetetraacetic acid (EDTA) and sheep mucosal swab samples were used. Sampling was proportionally distributed across the five regencies and included cattle within a 5 km radius of sheep farms, based on the relative cattle and sheep populations in each area.

### Data and sample collection

Clinical examinations and laboratory analyses of anticoagulated blood samples were performed on Bali cattle reared in proximity to sheep, using buffy coat isolation. Blood samples were collected from the jugular vein using sterile disposable 5 or 10 cc syringes and transferred into EDTA-anticoagulated Vacutainer tubes. Venipuncture was performed with the needle positioned parallel to the ground and the bevel facing upward, inserted at an angle of 30–45°. All procedures were carried out by trained veterinary technicians to minimize animal stress and ensure animal welfare. Sampling sites were prepared aseptically prior to blood collection to prevent conta-mination.

Primary data for risk factor analysis were obtained through structured questionnaires administered to cattle owners. The questionnaire was developed based on previously published epidemiological studies and field management guidelines. Content validity was assessed by three experts in veterinary epidemiology and ruminant management, who evaluated item clarity, relevance, and completeness. The questionnaire was pilot-tested on 10 cattle farmers outside the study area to ensure comprehensibility and consistency, and minor revisions were made before full implementation. The dependent variable was laboratory-confirmed MCF status as determined by nested PCR. Independent variables included cattle-level factors (breed, sex, age, cattle–sheep interaction, and clinical signs of MCF) and herd-level factors (herd size, management system, housing type, feed source, water source, sheep ownership, and cattle–sheep proximity). Secondary data were obtained through a review of relevant books, journals, and scientific articles.

### Laboratory investigation

DNA was extracted from the collected samples using Qiagen (Germany) QIAamp® DNA Mini Kit. MCF detection was subsequently performed using nested PCR. To ensure diagnostic reliability, the nested PCR protocol followed validation parameters recommended by the World Organization for Animal Health (WOAH) [5].

The assay targeting the OvHV-2 tegument protein gene has previously demonstrated high analytical sensitivity, with a detection limit of approximately 10–100 copies of target DNA, and 100% analytical specificity without cross-amplification with related herpesviruses, as reported by Baxter et al. [11] and subsequent studies. Method repeatability was confirmed in the present study through consistent amplification of the positive control across independent runs. Primer sequences used in the assay are presented in Table 1.

**Table 1:** Primer sequences used for the detection of ovine herpesvirus-2.

Primer name	Primer length	Primer sequence (5′–3′)
556	30 mer	5′-AGT CTG GGT ATA TGA ATC CAG ATG GCT CTC-3′
555	28 mer	5′-TTC TGG GGT AGT GGC GAG CGA AGG CTTC-3′
755	30 mer	5′-AAG ATA AGC ACC AGT TAT GCA TCT GAT AAA-3′

mer = nucleotide length expressed in bases.

DNA extraction involved preparation of buffy coat fractions from bovine blood collected in Onemed (Indonesia) EDTA–anticoagulated blood collection tubes, as well as sheep ocular, nasal, and vaginal swab samples. Extraction was performed using the Qiagen QIAamp® DNA Mini Kit according to the manufacturer’s instructions. PCR amplification was conducted using the Qiagen HotStarTaq® Plus Master Mix Kit.

To assess DNA quality and exclude PCR inhibition, all extracted samples were subjected to amplification of a housekeeping gene (β-actin) as an internal control. Only samples producing a clear β-actin band were included in the nested PCR analysis for OvHV-2. Samples failing internal control amplification were re-extracted and re-tested to ensure that negative OvHV-2 results reflected true negatives rather than poor DNA quality or inhibition.

**Figure 2:**
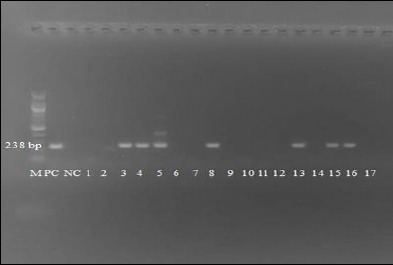
Agarose gel electrophoresis showing nested polymerase chain reaction amplification of ovine herpesvirus-2 from field samples. Lane M: DNA molecular weight marker, PC: positive control, NC: negative control, lanes 1–17: test samples.

The MF629 DNA sample, obtained from a confirmed SA-MCF case, was used as the positive control [11], while Qiagen Nuclease-free water served as the negative control. PCR amplification was performed using a Bio-Rad (USA) T100 Thermal Cycler. PCR amplification was performed in two stages: the first stage used primers 556 and 755, followed by a second stage using primers 556 and 555 [10–12]. Thermal cycling conditions for both stages included initial activation at 95°C for 5 min, followed by 35 cycles of denaturation at 94°C for 30 s, annealing at 64.4°C for 30 s, and extension at 72°C for 1 min, with a final extension at 72°C for 10 min. PCR products were held at 12°C until electrophoresis. Amplified products were visualized on 1.5% Invitrogen (USA) Agarose gels and documented using a SmartDoc™ Imaging Enclosure (USA) Ultraviolet transilluminator gel documentation system. Figure 2 shows agarose gel electrophoresis demonstrating nested polymerase chain reaction (PCR) amplification of ovine herpesvirus-2 in field samples. A limitation of this study was the absence of sequence confirmation, which could have provided definitive viral identification and phylogenetic insights. However, given the high genetic conservation of OvHV-2, sequencing would be particularly useful for tracing transmission sources [4].

### Statistical analysis

Questionnaire data and laboratory results were coded and systematically entered into Microsoft Excel. Animals with blood samples testing positive by nested PCR were classified as MCF-positive. A farm was considered positive if at least one animal tested positive by nested PCR. Individual-level prevalence was calculated as the proportion of positive animals among all tested animals, while farm-level prevalence was calculated as the proportion of positive farms among all surveyed farms.

Descriptive analyses, including frequencies and percentages, as well as univariate logistic regression, were performed using the Statistical Package for the Social Sciences version 27. Risk factor analysis was conducted in stages at the individual cattle and herd levels. Variables with p values <0.25 in chi-square or Fisher’s exact tests were retained for inclusion in the multivariable regression model.

## RESULTS

### Prevalence of MCF in Riau Province detected by nested PCR

Riau is a province located on the island of Sumatra. According to the Minister of Home Affairs Regulation No. 72 of 2019 dated October 8, 2019, Riau Province has an area of 87,023.66 km². Its territory stretches from the slopes of the Barisan Mountains to the Strait of Malacca, located between 01°05’00” South Latitude and 02°25’00” North Latitude, or between 100°00’00” East Longitude and 105°05’00” East Longitude. Bali cattle are a large ruminant livestock commodity widely cultivated in the Riau Province. In collaboration with the Veterinary Research Center (Ministry of Agriculture), the Animal Husbandry and Animal Health Service of Riau Province conducted MCF monitoring in 2021 across five districts (Bengkalis, Siak, Kampar, Pelalawan, and Rokan Hilir). Among the 50 sheep blood samples examined, 54% tested positive [6]. Building on these findings, the same districts were reinvestigated in the present study using cattle samples.

### Cattle population distribution

**Figure 3:**
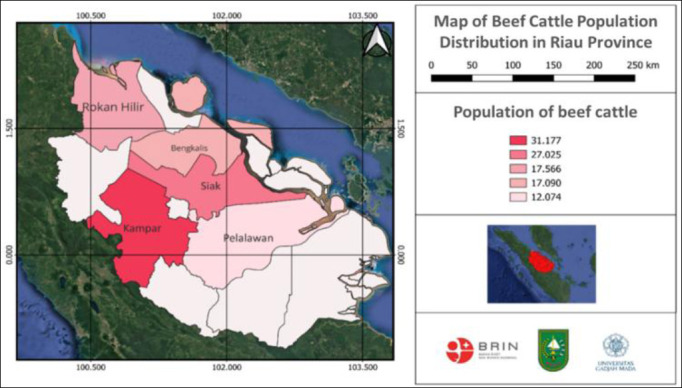
Map of the beef cattle population distribution in Riau Province, Indonesia.

Population data were obtained in 2022 from the Animal Husbandry and Animal Health Services Office of Riau Province. These data showed that Kampar District had the largest total cattle population, at 31,177. Rokan Hilir District had a total cattle population of 27,025. Siak District had a total cattle population of 17,566. Bengkalis and Pelalawan districts had total cattle populations of 17,090 and 12,074, respectively. Figure 3 shows the mapping results.

### Individual-level prevalence of MCF

The examination of 274 cattle across five districts in Riau Province showed that each district had a different number of positive MCF cases. Kampar District, with a total of 81 whole-blood cattle samples, had 10 positive samples, corresponding to a 12.35% (95% CI: 5.14–19.55) prevalence. In Pelalawan District, among 49 whole-blood samples from cattle, one was positive, yielding a positivity rate of 2.0% (95% CI: −1.96–6.04). Siak District, with a total of 72 bovine whole-blood samples, had nine positive samples, corresponding to 12.5% (95% CI: 4.81–20.19). Rokan Hilir District, with a total of 59 bovine whole-blood samples, had nine positive samples, corresponding to 15.3% (95% CI: 7.29–26.6). Bengkalis District, with a total of 13 bovine whole-blood samples, had three positive samples, corresponding to a 23.1% (95% CI: −0.76–46.92) positivity rate. Therefore, the total prevalence of MCF in Riau Province was 12.04%. More detailed results are presented in Table 2.

**Table 2:** Malignant catarrhal fever prevalence in Riau Province.

District	Cattle (n)	Pos	Prev (%)	95% CI	*Household cattle farms (n)	Pos	Prev (%)	95% CI	*Sheep (n)	Pos	Prev (%)	95% CI
Kampar	81	10	12.35	5.14–19.55	26	7	26.92	9.54–44.31	43	15	34.88	20.47–49.3
Pelalawan	49	1	2.04	–1.96–6.04	9	1	12.50	–12.00–37.00	26	15	57.69	38.33–77.06
Siak	72	9	12.5	4.81–20.19	20	6	28.57	8.77–48.37	45	19	42.22	27.63–56.82
Rohil	59	10	16.95	7.29–26.6	12	4	33.33	5.48–61.19	35	15	42.86	26.22–59.49
Bengkalis	13	3	23.08	–0.76–46.92	9	3	33.33	0.67–66.00	10	7	70.00	40.06–99.94
Total	274	33	12.04	8.18-15.90	76	21	27.63	17.51–37.75	159	71	44.65	36.90–52.40

Pos = number of positive samples, Prev = prevalence, CI = confidence interval.

### Farm-level prevalence of MCF

Of the 76 farms examined, 21 farms were confirmed positive for MCF, resulting in a total farm-level prevalence of 27.63% (95% CI: 17.51–37.75). The district exhibiting the highest prevalence was Rokan Hilir at 33.33% (95% CI: 5.48–61.19), followed by Bengkalis District at 33.33% (95% CI: 0.67–66.00). Siak District exhibited a prevalence rate of 28.57% (95% CI: 8.77–48.37). Kampar District demonstrated a prevalence rate of 26.92% (95% CI: 8.54–44.31). Pelalawan District recorded the lowest prevalence rate at 12.50% (95% CI: −12.00–37.00).

### Prevalence of OvHV-2 in sheep

The investigation identified OvHV-2 in sheep within the sample area. Among 159 analyzed sheep samples, the overall prevalence of OvHV-2 was 44.65% (95% CI: 36.90–52.40). Bengkalis District exhibited the highest prevalence at 70% (95% CI: 40.06–99.94). Pelalawan District exhibited a prevalence rate of 57.69% (95% CI: 38.33–77.06). This was followed by Rokan Hilir District at 42.86% (95% CI: 26.22–59.49), Siak District at 42.22% (95% CI: 27.63–56.82), and Kampar District at 34.88% (95% CI: 20.47–49.3).

### Clinical findings

Of the 274 cattle examined, 17 cattle showed symptoms consistent with MCF infection, including fever, weakness, bloody diarrhea, and nasal discharge. Of these 17 cattle with MCF-like symptoms, one cow from Sabak Auh Subdistrict, Siak District, was confirmed positive by nested PCR. Two weeks after sampling, the cow’s condition worsened and eventually resulted in death. Nested PCR testing confirmed that the remaining 32 MCF-positive cattle showed no clinical symptoms at the time of sampling.

### Individual-level factors associated with MCF in beef cattle

Analysis revealed that Bali cattle had a higher MCF positivity rate (13.1%) than non-Bali cattle (6.7%). Most cattle in both groups tested negative for MCF, with 86.9% negativity in Bali cattle and 93.3% negativity in non-Bali cattle. The chi-square test yielded a p-value of 0.336, indicating no significant association between cattle breed and MCF incidence (p > 0.05). However, Bali cattle tended to have a higher percentage of positive MCF cases. More detailed results are presented in Table 3.

**Table 3:** Results of univariate analysis between potential risk factors and the prevalence of malignant catarrhal fever in beef cattle in Riau Province, Indonesia.

Variable	Category	N	Number of positive	Prevalence (%)	95% CI	p-value	OR
Bali breed	Yes	229	30	13.10	8.72–17.48	0.336	–
	No	45	3	6.67	−0.70–14.04		
Sex	Male	197	9	11.69	4.47–18.91	1.000	–
	Female	77	24	12.18	7.60–16.76		
Age	≤2 years (Yes)	135	22	16.30	10.04–22.55	0.052	–
	≤2 years (No)	139	11	7.91	3.41–12.42		
	2–5 years (Yes)	108	5	4.63	0.65–8.61	0.004	0.239
	2–5 years (No)	166	28	16.87	11.15–22.58		
	>5 years (Yes)	31	6	19.35	5.22–33.49	0.184	–
	>5 years (No)	243	27	11.11	7.15–15.07		
Interaction with sheep	Yes	50	6	12.00	2.90–21.10	1.000	–
	No	244	27	12.05	7.96–16.15		
Clinical symptoms	Yes	18	1	5.88	−5.32–17.09	0.367	–
	No	257	32	12.45	8.41–16.50		
Herd size	≤4 animals	25	4	16.00	1.33–30.67	0.189	–
	>4 animals	51	17	33.33	−8.40–75.06		
Husbandry	Free-range (Yes)	9	3	33.33	0.67–66.00	0.477	–
	Free-range (No)	67	18	26.87	16.17–37.56		
	Confined (Yes)	33	11	33.33	17.00–49.67	0.475	–
	Confined (No)	43	10	23.26	10.48–36.03		
	Mixed (Yes)	34	7	20.59	6.79–34.38	0.328	–
	Mixed (No)	42	14	33.33	18.90–47.76		
Feed type	Grass-only	71	18	25.35	15.16–35.54	0.126	–
	Mixed feed	5	3	60.00	11.99–108.01		
Housing type	No shelter (Yes)	9	3	33.33	0.67–66.00	0.477	–
	No shelter (No)	67	18	26.87	16.17–37.56		
	Semi-permanent (Yes)	49	14	28.57	15.79–41.35	1.000	–
	Semi-permanent (No)	27	7	25.93	9.08–42.77		
	Permanent (Yes)	18	4	22.22	2.46–41.98	0.397	–
	Permanent (No)	58	17	29.31	17.49–41.13		
Forage source	Self-harvested	52	16	30.77	18.10–43.44	0.532	–
	Grazing	24	5	20.83	4.24–37.43		
Water source	Well (Yes)	57	15	26.32	14.78–37.85	0.882	–
	Well (No)	19	6	31.58	10.11–53.05		
	River (Yes)	11	2	18.18	−5.72–42.09	0.361	–
	River (No)	65	19	29.23	18.09–40.37		
	Pond (Yes)	8	4	50.00	12.96–87.04	0.141	–
	Pond (No)	68	17	25.00	14.63–35.37		
Sheep ownership	Yes	7	5	71.43	35.28–107.58	0.015	0.015
	No	69	16	23.19	13.16–33.22		
Sheep proximity	Present	70	20	28.57	17.91–39.23	0.467	–
	Absent	6	1	16.67	−16.00–49.33		
Cattle–sheep distance	<100 m (Yes)	13	7	53.85	25.64–82.05	0.027	4.083
	<100 m (No)	63	14	22.22	11.87–32.57		
	100–500 m (Yes)	18	4	22.22	2.46–41.98	0.397	–
	100–500 m (No)	58	17	29.31	17.49–41.13		
	500 m–1 km (Yes)	24	8	33.33	14.07–52.60	0.632	–
	500 m–1 km (No)	52	13	25.00	13.12–36.88		
	1–5 km (Yes)	14	2	14.29	−4.74–33.31	0.185	–
	1–5 km (No)	62	19	30.65	19.08–42.21		
	>5 km (Yes)	7	0	0.00	0.00–0.00	0.093	–
	>5 km (No)	69	21	30.43	19.50–41.37		

CI = confidence interval, OR = odds ratio. Univariate associations were assessed using chi-square or Fisher’s exact test, as appropriate. Odds ratios are shown only for categories with statistically significant associations.

Analysis of sex showed that females had an MCF positivity rate of 12.8%, while males had a positivity rate of 11.7%. Most cattle in both groups tested negative, with 87.8% negativity in females and 88.3% in males. The chi-square test yielded a p-value of 1.000, indicating no significant association between cattle sex and MCF incidence (p > 0.05). This finding indicates that sex was not a major factor influencing MCF incidence in this study.

Analysis of age showed that cattle aged ≤2 years had an MCF positivity rate of 16.3%, while cattle aged >5 years had a higher positivity rate of 19.4%. In contrast, cattle aged 2–5 years showed the lowest positivity rate at 4.6%. Most cattle in all age groups tested negative for MCF, with negativity rates of 83.7% in cattle aged ≤2 years, 95.4% in cattle aged 2–5 years, and 80.6% in cattle aged >5 years. The chi-square test yielded a p-value of 0.004 for the 2–5-year age group, indicating a significant association with reduced MCF incidence (p < 0.05). Cattle in this age group had a negative association with MCF incidence, with an OR of 0.239. In comparison, younger and older cattle showed higher MCF positivity, although these differences were not statistically significant.

Cattle without sheep contact showed an MCF positivity rate of 12.1%, while cattle with sheep contact showed a similar positivity rate of 12.0%. Most cattle in both groups tested negative for MCF, with 87.9% negativity in cattle without sheep contact and 88.0% in cattle with sheep contact. The chi-square test yielded a p-value of 1.000, indicating no significant association between sheep interaction and MCF incidence (p > 0.05).

Clinically symptomatic cattle showed an MCF positivity rate of 5.9%, while clinically asymptomatic cattle showed a higher positivity rate of 12.5%. Most cattle in both groups tested negative, with 94.1% negativity in symptomatic cattle and 87.5% in asymptomatic cattle. The chi-square test yielded a p-value of 0.420, indicating no significant association between clinical status and MCF incidence (p > 0.05).

### Herd-level factors associated with MCF in beef cattle

Analysis of herd size showed that farms with ≤4 cattle had 84% negative and 16% positive MCF results. Farms with more than four cattle showed 66.7% negative and 33.3% positive MCF results. The chi-square test yielded a p-value of 0.189, indicating no significant association between herd size and MCF incidence (p > 0.05). However, farms with larger herd sizes showed a higher proportion of MCF-positive cases.

Three cattle-rearing systems were identified: cattle released in pastures or palm plantations, cattle housed daily, and cattle released during the day and housed at night. Cattle released in pastures or palm plantations and cattle housed daily showed the same MCF positivity rate (33.3%). Cattle managed under the combined system showed an MCF positivity rate of 20.6%. The chi-square test yielded p > 0.05, indicating no significant association between management system and MCF incidence.

Three housing types were identified: no cage, semi-permanent cage, and permanent cage. Cattle kept without cages showed an MCF positivity rate of 33.3%, those in semi-permanent cages showed 28.6%, and those in permanent cages showed 22.2%. The chi-square test yielded p > 0.05, indicating no significant association between housing type and MCF incidence, although higher positivity was observed in cattle without cages.

Analysis of feed type showed that cattle fed fresh grass had an MCF positivity rate of 25.4%, while cattle fed a combination diet had a positivity rate of 60%. Most cattle fed fresh grass tested negative (74.6%), while only 40% of cattle fed a combination diet tested negative. The chi-square test yielded a p-value of 0.126, indicating no significant association between feed type and MCF incidence (p > 0.05). More detailed results are presented in Table 3.

Analysis of grass source showed that cattle fed grass collected from the cowshed had an MCF positivity rate of 30.8%, while released cattle had a lower positivity rate of 20.8%. Most released cattle tested negative (79.2%), compared with cattle fed grass from cowsheds (69.2%). The chi-square test yielded a p-value of 0.532, indicating no significant association between grass source and MCF incidence (p > 0.05). However, cattle fed grass sourced from cowsheds tended to show higher MCF positivity.

Three drinking water sources were identified: wells, rivers, and puddles. Cattle drinking well water showed an MCF positivity rate of 26.3%, cattle drinking river water showed 18.2%, and cattle drinking from puddles showed the highest positivity rate at 50%. The chi-square test yielded p > 0.05, indicating no significant association between water source and MCF incidence. However, cattle drinking from puddles had a higher risk of MCF positivity.

Analysis of sheep ownership showed that cattle not reared together with sheep had an MCF positivity rate of 23.2%, while cattle reared together with sheep showed a much higher positivity rate of 71.4%. Most cattle not reared with sheep tested negative (76.8%), whereas only 28.6% of cattle reared with sheep tested negative. The chi-square test yielded a p-value of 0.015, indicating a significant association between sheep ownership and MCF incidence (p < 0.05), with an OR of 8.218.

Analysis of the distance between cattle and sheep showed that cattle located within 100 m of sheep had the highest MCF positivity rate (53.8%). In contrast, all cattle located more than 5 km away tested negative (100%). Cattle located 100–500 m away showed a positivity rate of 22.2%, cattle located 500 m–1 km away showed 33.3%, and cattle located 1–5 km away showed 14.3%. The chi-square test yielded a p-value of 0.027 for cattle located within 100 m, indicating a significant association between close proximity to sheep and MCF incidence (*p* < 0.05), with an OR of 4.083.

### Multivariate logistic regression analysis

Multivariate logistic regression analysis identified two significant variables associated with MCF incidence in beef cattle in Riau Province: cattle age and sheep ownership (Table 4). The incidence of MCF was reduced in cattle aged 2–5 years (OR = 0.245; 95% CI: 0.091–0.656). In contrast, concurrent rearing of cattle and sheep significantly increased the likelihood of MCF occurrence in cattle (OR = 8.958; 95% CI: 1.541–52.083).

**Table 4:** Results of the multivariate logistic regression analysis for potential risk factors of malignant catarrhal fever in beef cattle in Riau Province, Indonesia.

Factor	Category	B	S.E.	Sig.	OR	95% CI (Lower–Upper)
Age	2–5 years	−1.408	0.504	0.005	0.245	0.091–0.656
Sheep ownership	Yes	2.193	0.898	0.015	8.958	1.541–52.083

B = regression coefficient, S. E. = standard error, Sig. = significance level (p-value), OR = odds ratio, CI = confidence interval. Odds ratios were obtained from the multivariate logistic regression model.

## DISCUSSION

### Molecular evidence of OvHV-2 circulation in Riau Province

This study provides updated molecular evidence of OvHV-2 circulation in beef cattle in Riau Province, Indonesia. The overall prevalence of 12.04% observed in this study demonstrates that OvHV-2 is enzootic in the region, although the infection is likely underrecognized because it is predominantly subclinical in nature. This finding is consistent with previous reports from other provinces in Indonesia, where the prevalence of OvHV-2 ranges from low to moderate depending on animal species, management systems, and diagnostic approaches used.

### Etiology and diversity of MCF-associated viruses

MCF is caused by a group of herpesviruses belonging to the genus Macavirus within the subfamily Gammaherpesvirinae. At least ten viral types have been identified within this group, six of which are pathogenic under natural conditions. These pathogenic viruses include alcelaphine herpesvirus 1 (AlHV-1) in the wildebeest reservoir, OvHV-2 in the sheep reservoir, caprine herpesvirus 2 (CpHV-2) in the goat reservoir, ibex-MCF virus in the ibex reservoir, white-tailed deer MCF virus in the white-tailed deer reservoir, and alcelaphine herpesvirus 2–like virus in the Jackson hartebeest reservoir [13, 14].

AlHV-1 carried by wildebeest is the causative agent of WA-MCF. This form of MCF represents a serious problem in Africa and in zoological collections, such as zoos, where clinically susceptible animals are kept in proximity to wild animals like wildebeest. OvHV-2 carried by sheep causes SA-MCF, which is a major global problem, particularly for highly susceptible species such as Bali cattle, deer, bison, pigs, giraffes, and various species of hoofed animals maintained in zoological collections [14].

### Pathogenesis and host–virus interaction

Herpesviruses are highly adapted to their hosts, and individuals with compromised immune systems are more susceptible to severe infections. Primary systemic infection typically occurs through cell-associated viremia, followed by a latent phase during which the virus may occasionally reactivate. Herpesviruses use a variety of mechanisms to regulate and evade the host immune response [15].

OvHV-2 has a prolonged incubation period ranging from 2 to 10 weeks. The virus initially replicates in lung tissue and subsequently infects lymphocytes and endothelial cells. This process induces dysfunction in immunoregulatory pathways within the host immune system [16].

### Comparison with regional and international prevalence studies

Research on MCF prevalence has been conducted in many countries worldwide; however, data from Indonesia remain limited. Previous studies have reported variable prevalence estimates. In India, a 2013 study using nested PCR examined 356 sheep peripheral blood leukocyte samples and reported an MCF prevalence of 24.4% [17]. In Pakistan, a 2021 nested PCR study using blood samples from cattle, buffalo, goats, and sheep reported prevalence rates of 9%, 6.5%, 11%, and 13%, respectively [18].

In the present study, conducted in Riau Province using nested PCR, cattle blood samples showed an MCF prevalence of 12.04%, while sheep mucosal swab samples showed a prevalence of 44.65%.

### Breed-related susceptibility to MCF

The results of this study showed no statistically significant difference in MCF incidence between cattle breeds. However, there was a tendency for Bali cattle to have a higher proportion of MCF-positive cases compared with non-Bali cattle. Daniels *et al*. [10] reported that animals susceptible to MCF include Bali cattle, crossbred Bali cattle, buffalo, Ongole cattle, and Brahman cattle.

If the population of cattle in contact with OvHV-2–carrying sheep is high, the probability of MCF infection increases automatically. Bali cattle are known to be susceptible to MCF, and during outbreaks, infection typically occurs through viral secretions from pregnant or recently lambed sheep [19]. Hussain et al. [20] reported that Bali cattle, bison, and deer are more susceptible to SA-MCF than *B. indicus* and *B. taurus*. Other studies also reported no statistically significant association between breed differences and MCF incidence in cattle [21, 22].

### Sex as a risk factor for MCF

Studies that specifically evaluate sex as a risk factor for MCF infection are limited. In this study, sex was not significantly associated with MCF incidence. Curtis [23] reported no strong statistical evidence of an association between sex and MCF incidence, although females had a 1.5-fold higher risk of developing the disease than males. Murid *et al*. [21] in Egypt and Raffaele *et al*. [22] in Italy also reported that sex did not influence the occurrence of MCF in cattle.

### Age-related susceptibility to MCF

This study showed significant findings for cattle aged 2–5 years, indicating that this age group had a lower risk of MCF infection. In contrast, younger cattle (≤2 years) and older cattle (>5 years) had a higher risk of infection, although these differences were not statistically significant. These results are consistent with findings reported by Murid *et al*. [21], who observed a tendency toward higher infection rates in young cattle.

In this study, cattle aged 1–3 years were the most susceptible group. This increased susceptibility may be explained by the loss of maternal immunity during this period, which increases vulnerability to OvHV-2 infection. Cattle older than 3 years generally show lower susceptibility to MCF, possibly due to the development of stronger immune responses or partial resistance to OvHV-2.

Curtis [23] reported that age was a significant risk factor for MCF, with calves aged 6 months or less at the highest risk. Cattle with immature immune systems, particularly young calves, are therefore more susceptible to MCF. Although MCF can occur in cattle older than 2 years, several studies have shown that the risk of infection in this age group is not consistently higher than in younger cattle.

### Sheep interaction and transmission dynamics

The interaction between sheep and cattle showed no significant association with MCF incidence in this study. Previous studies have suggested that interactions between sheep and cattle increase the risk of OvHV-2 transmission, primarily through aerosols generated by nasal secretions of infected or carrier sheep [10, 21, 24, 25]. An MCF outbreak in Spain occurred after calves were temporarily housed in close proximity to sheep pens [26].

Differences between the results of this study and those of previous studies are likely due to cattle-rearing practices in Riau Province. Most cattle in Riau are raised semi-intensively or extensively and are often released into oil palm plantations. Under these conditions, cattle owners may not observe direct interaction between cattle and sheep. This is supported by questionnaire data indicating that 224 of 274 cattle were never observed interacting with sheep.

### Clinical presentation and subclinical infection

This study also showed no significant association between clinical symptoms in cattle and MCF incidence. MCF is clinically characterized by high fever, anorexia, conjunctivitis, and mucosal inflammation, which may progress to neurological disorders and death. Clinical signs include excessive nasal and ocular discharge, hypersalivation, corneal opacity, nasal and oral ulcers, lymphadenopathy, respiratory distress, and diarrhea. Pathological findings commonly include inflammation of multiple organs, vasculitis, and mononuclear cell infiltration [10, 21, 25–33].

In some cases, young cattle showed resilience following symptomatic treatment, highlighting the importance of early intervention. OvHV-2 can persist in the bloodstream for several weeks after infection, indicating a prolonged viremic phase [10, 21, 25–33].

Subclinical or asymptomatic MCF infection has been widely reported. Asymptomatic cattle infected with MCF carry OvHV-2 without showing clinical signs [34, 35]. Cattle and buffalo are considered dead-end hosts because they do not transmit the virus to other animals. Infection in cattle and buffalo is often subclinical, although they may still carry OvHV-2 acquired through contact with infected sheep or goats [18].

Asymptomatic MCF in Bali cattle is difficult to detect clinically because obvious signs such as high fever or corneal opacity are absent. Nevertheless, immunological and histopathological changes may be present, including lymphocyte proliferation and mononuclear cell infiltration in lymphoid tissues, particularly lymph nodes, indicating an immune response to the virus [36, 37].

Headley *et al*. [38] suggested that OvHV-2 can establish subclinical or latent infection in cattle, which often remains undiagnosed if detection relies solely on clinical signs. Factors contributing to subclinical infection include low viral load, latency, absence of free virus propagation, environmental stress, co-infection with other pathogens, and indirect transmission. Another study reported that approximately 70% of cattle showed evidence of OvHV-2 infection by PCR or serology without clinical signs of MCF. As a gammaherpesvirus, OvHV-2 establishes lifelong latency in lymphocytes and may reactivate under conditions of reduced immunity [39].

### Farm-level risk factors and environmental exposure

Studies on farm-level risk factors showed mixed results. No significant association was found between herd size and MCF incidence, although farms with more livestock showed a higher proportion of positive cases. Daniels *et al*. [10] reported that high livestock density and the presence of multiple livestock species increase the risk of MCF. Environmental factors such as overcrowding can induce stress, reduce immune function, and increase susceptibility to infection [25].

The increase in MCF incidence on farms is not due to transmission among cattle. Headley *et al*. [30] reported that cattle, deer, bison, and buffalo are not part of the viral transmission cycle and therefore cannot transmit the virus to one another. This conclusion is supported by other studies indicating that no virus shedding occurs in cattle or buffalo, confirming their role as dead-end hosts [16, 18]. Consequently, proximity to reservoir hosts is the key determinant of MCF incidence [40].

### Husbandry, feeding, and water-related factors

No significant association was found between MCF incidence and husbandry type or pen type. However, the proportion of MCF-positive cattle tended to be higher on farms without cages. Most cattle in Riau are raised semi-intensively or extensively and are released into oil palm plantations, increasing potential exposure to OvHV-2. Curtis [23] reported MCF cases in calves that were not kept in cages, likely due to indirect exposure through aerosols, mucosal secretions, or contaminated fomites. Power *et al*. [39] also reported virus transmission on cattle farms located approximately 70 m from sheep pens.

Feed type and feed source were not significantly associated with MCF incidence. However, an interesting observation was made in the Tapung area of Kampar District. Fourteen cattle samples were collected, including three cattle released in oil palm plantations near sheep grazing areas and 11 cattle housed 500 m to 1 km from sheep grazing sites. Eight of the 14 samples tested positive for MCF, with seven positives originating from cattle housed within 500 m to 1 km of sheep. Feed tracing revealed that the grass used for feeding was harvested from oil palm plantations shared with sheep grazing. Mixed grazing practices, while improving pasture utilization, increase the risk of OvHV-2 exposure [18, 41].

The drinking water source also showed no statistically significant association with MCF incidence. Nevertheless, cattle drinking water from puddles tended to show a higher risk. Water sources may become contaminated by sheep nasal secretions containing OvHV-2. MCF viruses released through aerosols, mucosal secretions, or fomites can contaminate the environment and facilitate indirect transmission [20, 23]. Headley *et al*. [34] noted that MCF can occur through indirect contact, possibly involving mechanical vectors or environmental spread. Limited access to drinking water may further increase stress and susceptibility to disease [33].

### Impact of sheep co-rearing and spatial proximity

The results of this study demonstrated a significant association between sheep co-rearing and MCF incidence. Cattle raised in close proximity to sheep had a higher risk of MCF than those not kept near sheep. These findings are consistent with previous studies reporting increased MCF incidence when cattle and sheep are raised together [13, 17, 33]. Zakharova *et al*. [28] and Timurkan *et al*. [37] emphasized that close contact with sheep, which serve as natural reservoirs, significantly increases MCF risk. Several studies recommend avoiding cohabitation of sheep with other hoofed animals, particularly during lambing, to reduce SA-MCF outbreaks [20, 21, 31].

Distance analysis showed that cattle within 100 m of sheep had a higher risk of MCF than those farther away. Taus *et al*. [43] demonstrated that nasal secretions from sheep can transmit infection through the respiratory route. MCF transmission has been reported at distances of 1–5 km, although the exact mechanism remains unclear. Headley *et al*. [38] reported MCF infection in bison kept within 1 km of sheep, while Power *et al*. [39] reported transmission at distances as short as 70 m. Cattle within 100 m of sheep pens have also been reported to develop MCF [44].

The findings of Timurkan *et al*. [42] align with studies by Wiyono and Damayanti [36] and Li *et al*. [45], which emphasized the importance of distance between reservoir and susceptible animals. The closer the distance, the greater the likelihood of exposure. Studies from India and Kashmir further support this observation, showing higher MCF incidence in areas where cattle are kept close to sheep or wildebeest [20].

This study highlights the importance of strengthening One Health–based management by improving integration of cattle and sheep production systems and considering wildlife exposure in mixed-farming environments. Preventing spillover through spatial planning, reduced cross-species contact, and improved farmer awareness is essential. Active molecular surveillance remains critical due to the silent nature of OvHV-2 infection. Future studies should evaluate temporal transmission patterns, sheep shedding dynamics, and environmental drivers of exposure, while developing practical, community-level interventions suitable for smallholder systems.

## CONLUSION

This study provides robust molecular evidence confirming the endemic circulation of OvHV-2 in cattle in Riau Province. Using nested PCR, an overall cattle-level prevalence of 12.04% was detected, while a substantially higher prevalence was observed in sheep, confirming their role as the primary reservoir. Although most infected cattle were clinically asymptomatic, OvHV-2 infection was widely distributed across districts and farms, highlighting the silent epidemiological burden of MCF in the region. Multivariable analysis identified cattle age and sheep co-rearing as significant determinants of infection risk: cattle aged 2–5 years had a lower likelihood of infection, whereas cattle kept with sheep had a markedly increased risk, as reflected by elevated OR values.

The findings underscore the need for targeted disease prevention strategies in mixed-ruminant production systems. Practical control measures should prioritize spatial separation between cattle and sheep, particularly during periods of increased viral shedding in sheep. Improved farm-level biosecurity, informed spatial planning, and farmer education on indirect transmission routes are critical to reducing OvHV-2 exposure. Given the predominantly subclinical nature of infection, reliance on clinical surveillance alone is insufficient, and routine molecular monitoring using PCR-based methods is strongly recommended.

A major strength of this study lies in its integrated approach, combining molecular diagnostics with detailed individual- and herd-level risk factor analysis across multiple districts. The use of nested PCR enhanced diagnostic sensitivity, enabling the detection of subclinical infections that would otherwise remain unrecognized. Additionally, the inclusion of spatial proximity and management variables provides context-specific evidence relevant to smallholder and semi-intensive cattle systems typical of the region.

Despite these strengths, several limitations should be acknowledged. Viral sequencing was not performed, which restricted confirmation of genetic relatedness and hindered assessment of transmission pathways. The cross-sectional design also limits inference on temporal dynamics and causality. Furthermore, environmental sampling was not included, which may have provided additional insight into indirect transmission routes.

Future research should incorporate longitudinal study designs to better elucidate temporal patterns of OvHV-2 transmission and shedding dynamics in sheep. Molecular characterization and sequencing of detected viruses would strengthen understanding of viral sources and epidemiological links. Expanding surveillance to include environmental samples and wildlife interfaces would further support a One Health perspective. Development and evaluation of practical, community-level intervention models tailored to mixed-farming systems are also warranted.

In conclusion, this study demonstrates that MCF caused by OvHV-2 represents a persistent yet underdiagnosed threat to cattle production in Riau Province. The strong association with sheep co-rearing and close spatial proximity emphasizes the importance of integrated management strategies. Strengthening molecular surveillance, improving farm management practices, and enhancing stakeholder awareness are essential steps to mitigate the impact of this disease and safeguard livestock health and productivity in mixed-ruminant systems.

## AUTHORS’ CONTRIBUTIONS

AY: Conceived and designed the study, coordinated field sampling, and contributed to data interpretation. DAW: Aupervised the overall research project, guided methodological development, and critically revised the manuscript. AW and RD: Performed the laboratory analyses, including DNA extraction and nested PCR. FS and DA: Assisted with sample collection, farm coordination, and field data recording. AY and DAW: Analyzed the data and drafted the manuscript. All authors have read and approved the final version of the manuscript.

## COMPETING INTERESTS

The authors declare that they have no competing interests.

## PUBLISHER’S NOTE

Veterinary World remains neutral with regard to jurisdictional claims in the published maps and institutional affiliations.
